# *Plasmodium vivax* Liver and Blood Stages Recruit the Druggable Host Membrane Channel Aquaporin-3

**DOI:** 10.1016/j.chembiol.2020.03.009

**Published:** 2020-06-18

**Authors:** Dora Posfai, Steven P. Maher, Camille Roesch, Amélie Vantaux, Kayla Sylvester, Julie Péneau, Jean Popovici, Dennis E. Kyle, Benoît Witkowski, Emily R. Derbyshire

**Affiliations:** 1 Department of Molecular Genetics and Microbiology, Duke University Medical Center, 213 Research Drive, Durham, NC 27710, USA; 2 Center for Tropical and Emerging Global Diseases, University of Georgia, 500 D.W. Brooks Dr, ste 370, Athens, GE 30602, USA; 3 Malaria Molecular Epidemiology Unit, Pasteur Institute in Cambodia, Phnom Penh 12201, Cambodia; 4 Chemistry Department, Duke University, 124 Science Drive, Durham, NC 27708, USA

**Keywords:** *Plasmodium*, *vivax*, aquaporin, hypnozoite, antimalarial

## Abstract

*Plasmodium vivax* infects hepatocytes to form schizonts that cause blood infection, or dormant hypnozoites that can persist for months in the liver before leading to relapsing blood infections. The molecular processes that drive *P*. *vivax* schizont and hypnozoite survival remain largely unknown, but they likely involve a rich network of host-pathogen interactions, including those occurring at the host-parasite interface, the parasitophorous vacuole membrane (PVM). Using a recently developed *P*. *vivax* liver-stage model system we demonstrate that host aquaporin-3 (AQP3) localizes to the PVM of schizonts and hypnozoites within 5 days after invasion. This recruitment is also observed in *P*. *vivax*-infected reticulocytes. Chemical treatment with the AQP3 inhibitor auphen reduces *P*. *vivax* liver hypnozoite and schizont burden, and inhibits *P*. *vivax* asexual blood-stage growth. These findings reveal a role for AQP3 in *P*. *vivax* liver and blood stages and suggest that the protein may be targeted for therapeutic treatment.

## Highlights

• Host aquaporin-3 (AQP3) is recruited to *P. vivax* hypnozoites and schizonts
• The AQP3 inhibitor auphen inhibits *P. vivax* hypnozoites and schizonts
• Host AQP3 is recruited to *P. vivax*-infected erythrocytes derived from patient samples
• Auphen inhibits blood stages of clinical *P. vivax* isolates

## Graphical Abstract

**Figure:**
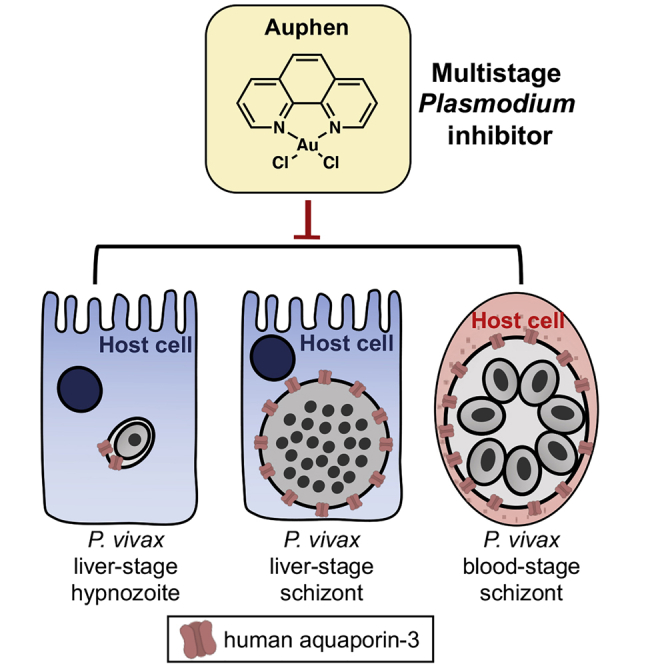

## Teaser

Using a recently developed *Plasmodium vivax* liver-stage model, Posfai et al. describe the recruitment of human aquaporin-3 to the host-parasite interface during the liver and blood stages. Furthermore, they demonstrate that an aquaporin-3 inhibitor, auphen, is effective against multiple parasite stages, including hypnozoites.

## Introduction

The *Plasmodium* parasite that causes malaria led to over 200 million cases of the disease in 2018 (WHO, 2019). Among the parasite species that infect humans, *P*. *vivax* is the most widely dispersed and is primarily responsible for relapse infections (Battle et al., 2019). Transmission occurs when sporozoites are injected into a new host by the bite of an infected *Anopheles* mosquito. Individual sporozoites migrate to the liver, invade a hepatocyte, and form either a liver schizont or hypnozoite (Mueller et al., 2009). Over 9–12 days, schizonts produce thousands of daughter merozoites that infect and propagate within reticulocytes, causing malaria. Liver schizonts have also been shown to produce merozoites expressing biological markers for gametocyte development, indicating possible direct transmission from the liver after a single round of infection of liver merozoites into reticulocytes (Roth et al., 2018a, Roth et al., 2018b). Such a scenario would indicate “silent” transmission occurring before malaria symptoms and treatment with antimalarials. Alternatively, over the first several days of liver infection the sporozoite may form a hypnozoite which grows slightly before becoming biologically quiescent (Krotoski et al., 1982, Mikolajczak et al., 2015). With many biological processes inactive, these forms are insensitive to most antimalarials except 8-aminoquinolones, which are contraindicated in many malaria-vulnerable populations, including pregnant women, younger children, and individuals with glucose-6-phosphate dehydrogenase deficiency (Baird et al., 2018). Taken together, the *P*. *vivax* liver stage is an important unmet area of therapeutic intervention.

Although studies of *P*. *vivax* liver-stage biology and drug discovery have been recently reported (Gural et al., 2018, Roth et al., 2018a), progress is slowed by limited access to *P*. *vivax*-infected mosquitoes and the complexities of *in vitro* culturing systems. Conversely, because of ubiquitous use in the malaria research community, much of our current understanding of liver-stage biology stems from studies that utilize the mouse-infective *P*. *berghei* or *P*. *yoelii* models (Langhorne et al., 2011). Although these systems have enabled large-scale drug discovery campaigns (Antonova-Koch et al., 2018, Derbyshire et al., 2014) and diverse molecular studies to understand biology, the parasites do not form hypnozoites (Orjuela-Sanchez et al., 2018). Thus, a large gap in our understanding of hypnozoite biology exists. In addition, many of the biological mechanisms observed in mouse-infective *Plasmodium* have yet to be confirmed in the *P*. *vivax* liver stage. Revealing the molecular processes that drive hypnozoite persistence and activation, including possible host-pathogen interactions, could advance our understanding of this obscure and elusive parasite form. Once revealed, these processes may be targeted for small-molecule disruption to strengthen the current chemical arsenal against *Plasmodium*.

As an obligate intracellular parasite, *Plasmodium* relies on various host processes for proper nutrient acquisition, growth, and maturation (Dellibovi-Ragheb et al., 2018, Posfai et al., 2018, Sá E Cunha et al., 2017). During hepatocyte and blood cell invasion *Plasmodium* generates a parasitophorous vacuole membrane (PVM) through invagination of the host cell membrane (Nyboer et al., 2018). This PVM serves as the host-pathogen interface throughout infection and is requisite for *Plasmodium* growth and development. Using a *P*. *berghei* model, we previously identified the host water and small-molecule channel aquaporin-3 (AQP3) as essential to parasite development in hepatoma cells (Posfai et al., 2018). This human protein is recruited to the PVM in liver-stage *P*. *berghei* schizonts and blood-stage *P*. *falciparum* schizonts, and is thought to have a role in the movement of water or nutrients between the parasite and its host cell. To understand if similar mechanisms occur in *P*. *vivax* we utilized a recently developed, primary human hepatocytes (PHH)-based 384-well *P*. *vivax* liver-stage culture platform to characterize AQP3 recruitment in *P*. *vivax* liver forms (Roth et al., 2018a). Here, we demonstrate that host AQP3 is recruited to *P*. *vivax* liver schizonts and hypnozoites shortly after hepatocyte invasion. In addition, this recruitment is observed in *P*. *vivax*-infected reticulocytes. The AQP3 inhibitor auphen reduces *P*. *vivax* parasite load in *ex vivo* culture of clinical blood isolates as well as liver schizonts and hypnozoites. Our findings highlight the critical role of host AQP3 to *Plasmodium* and suggest that its function may be modulated to facilitate a target-based approach for disease-control efforts. Furthermore, this work progresses a hypothesis generated from the *P*. *berghei* liver-stage model system to multiple stages and forms of *P*. *vivax*, a medically relevant species.

## Results

### Host Membrane Protein AQP3 Localizes to the *P*. *vivax* Liver-Stage Parasitophorous Vacuole

**Figure 1:**
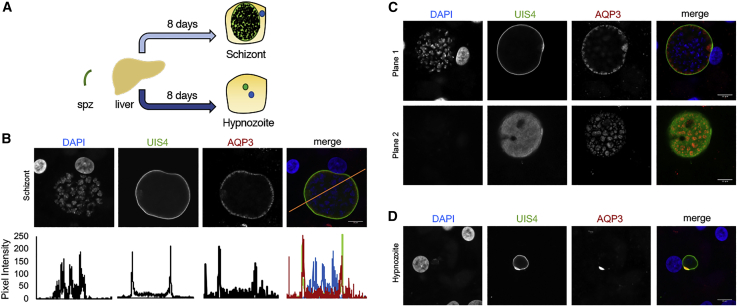
Human AQP3 Is Recruited to *P*. *vivax* Schizonts and Hypnozoites (A) Schematic of *P*. *vivax* sporozoite infection of PHH to produce liver schizonts or hypnozoites. (B) Representative confocal images of a *P*. *vivax* liver schizont (top) day 8 post-infection into PHH. Cells were stained with DAPI (blue), *Pv*UIS4 (green), and *Hs*AQP3 (red). Colocalization analysis of *Pv*UIS4 and *Hs*AQP3 (bottom). (C) Representative confocal images of a *P*. *vivax* liver schizont on day 8 post-infection of PHH at two different focal planes. (D) Human AQP3 localization in a representative *P*. *vivax* hypnozoite day 8 post-infection of PHH. Cells were stained with DAPI (blue), *Pv*UIS4 (green), and *Hs*AQP3 (red). Scale bar, 10 μm.

Few host factors are known to be important for *P*. *vivax* liver-stage development despite compelling evidence that *Plasmodium* depends on its host throughout infection. Human AQP3, a channel that fluxes water, glycerol, and other small neutral molecules (Carbrey and Agre, 2009, Laforenza et al., 2016), is upregulated after *P*. *berghei* infection of human hepatocytes and plays a critical role in parasite development (Posfai et al., 2018). We hypothesized that AQP3 may also have an important function in *P*. *vivax* liver-stage schizont and hypnozoite biology. To test this, we utilized *P*. *vivax* sporozoites harvested from freshly dissected *Anopheles* mosquitoes to infect PHHs (Roth et al., 2018a). For initial tests, we evaluated liver forms 8 days post infection (dpi), when parasites can be distinguished as large, actively replicating schizonts or small dormant hypnozoites based on their size and lack of nuclear replication (Mikolajczak et al., 2015) (Figure 1A). Based on immunofluorescence microscopy, host AQP3 is recruited to mature *P*. *vivax* liver schizonts (Figure 1B). Controls were completed with primary and secondary antibodies with uninfected and infected cells (HepG2, HeLa, HuH7, and PHH) to establish characteristics of background staining. At 8 dpi, no AQP3 signal above background was observed in uninfected PHH, but protein was detected in *P*. *vivax*-infected PHH (Figure S1A). We observed colocalization of AQP3 with *Plasmodium* UIS4 (upregulated in infective sporozoites gene 4), a secreted parasite protein that is incorporated to the PVM during early liver-stage infection (Mueller et al., 2005). Interestingly, AQP3 does not uniformly distribute throughout the PVM, in contrast to *Pv*UIS4, which continuously and evenly associates with the vacuole. Confocal images at various planar fields (Figure 1C; Video S1) reveal a patterned or punctate AQP3 localization. Since AQP3 serves as a water and solute channel in humans, we compared localization to that of the nutrient-permeable channel *Plasmodium* EXP2, a PVM component (Garten et al., 2018, Hall et al., 1983). We observed patterned staining at the PVM for *Pv*EXP2 in infected cells, similar to previous reports (Roth et al., 2018a). Despite the similar phenotype, AQP3 did not strictly localize to *Pv*EXP2 (Figure S1B) suggesting a distinct function. At 8 dpi, AQP3 was also associated with *P*. *vivax* hypnozoites (Figures 1D; Video S2), but staining was not uniform around the PVM in every infection. AQP3 was primarily limited to PVM regions where *Pv*UIS4 staining was most prominent as a small crescent. The known role of AQP3 in human cells, and its recruitment to *P*. *vivax* liver forms, supports a possible function as a channel- or vacolue-resident protein associated with the PVM.

Video S1. Host AQP3 Is Recruited to the PVM in *P. vivax* Schizonts, Related to Figure 1 *P. vivax* liver-stage schizont, related to Figure 1. z stack video of *Hs*AQP3 localization on day 8 of infection. *Hs*APQ3 in red; *Pv*UIS4 in green is labeling the *Plasmodium* PVM; DAPI in blue is labeling the nuclei.

Video S2. Host AQP3 Is Recruited to the PVM in *P. vivax* Hypnozoites, Related to Figure 1 *P. vivax* liver-stage hypnozoite, related to Figure 1. z stack video of *Hs*AQP3 localization on day 8 of infection. *Hs*APQ3 in red; *Pv*UIS4 in green is labeling the *Plasmodium* PVM; DAPI in blue is labeling the nuclei.

**Figure 2:**
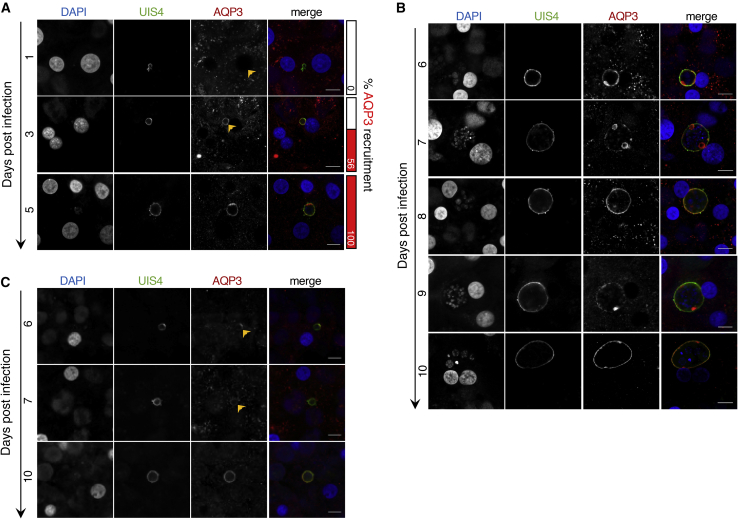
*P*. *vivax* Schizonts and Hypnozoites Recruit Human AQP3 at Early Stages of Infection (A) Time course of human AQP3 localization during early stages of *P*. *vivax* infection of PHH. Percentage of infected cells with detectable AQP3 localization shown (right, red columns). (B) Time course of human AQP3 localization in *P*. *vivax* schizonts in PHH. (C) Time course of human AQP3 localization in *P*. *vivax* hypnozoites in PHH. Cells were stained with DAPI (blue), *Pv*UIS4 (green), and *Hs*AQP3 (red). Representative confocal images shown (n = 15–38). Scale bar, 10 μm.

To understand how AQP3 expression and recruitment is manipulated by *P*. *vivax*, we completed a time course study where parasite-infected PHH were fixed every day for 11 days. Before 5 dpi, infections that will go on to become actively replicating forms versus hypnozoites are not readily distinguishable by size alone (Figures 2A and S2A). During this early window of *P*. *vivax* liver-stage development, *Pv*UIS4 localizes to the PVM but we observed no AQP3 recruitment at 1 dpi (n = 4). The earliest detection of AQP3 recruitment was 2 dpi. Microscopy analysis demonstrates robust *Pv*UIS4 localization to the PVM by 2 dpi, but only 19% of *P*. *vivax*-infected cells exhibited AQP3 recruitment (n = 16) (Figure S3A). AQP3 recruitment then continued progressively until 5 dpi, when all *P*. *vivax* infections had AQP3 at the PVM (Figures 2A and S2A). After 5 dpi, hypnozoites and schizonts are distinguishable by their size and nuclei number (Gualdrón-López et al., 2018). *P*. *vivax* liver schizonts then maintained AQP3 at the PVM throughout the study (5–11 dpi). In some liver schizonts, a higher AQP3 density was observed in puncta (Figures 2B, S2B, and S3B). Throughout infection, AQP3 localization correlates with *Pv*UIS4 staining in schizonts and hypnozoites (Figures S3B–S3D). The colocalization of AQP3 with *Pv*UIS4 (>50%) suggests incorporation of AQP3 into the PVM.

Hypnozoites are characterized by their small size and single nucleus, but chemical treatment has become a useful approach to enrich for hypnozoites in a population of *P*. *vivax*-infected hepatcytes. This method uses a phosphatidylinositol 4-kinase inhibitor (PI4Ki), such as MMV390048, that selectively clears mature schizonts when added on or after 5 dpi, but does not inhibit hypnozoites (Gural et al., 2018, Zeeman et al., 2016). To better understand when hypnozoites become PI4Ki insensitive we performed a time course study in which 1 μM MMV390048 was added to *P*. *vivax-*infected PHHs with treatment beginning each dpi from days 1 to 7. All wells were then fixed and stained on 8 dpi and high-content imaging (HCI) was used to quantify number of hypnozoites, number of schizonts, and schizont growth area in each well. The experiment was performed with two arms, one using a single addition of PI4Ki at each time point, and the other with two additional treatment days after the initial treatment, to understand the treatment coverage needed for PI4Ki-elimination of liver schizonts. PI4Ki-insensitive hypnozoites began appearing at 3 dpi while the full population was not established until 6 dpi, with the bulk of the population appearing at 5 dpi. Furthermore, we found a single day of PI4Ki treatment was sufficient to kill liver schizonts (Figure S4A). We then used MMV390048 treatment at 5 dpi to obtain *P*. *vivax*-infected PHH cultures with only hypnozoites and found that 100% of hypnozoites had *Hs*AQP3 recruitment by 6 dpi. This recruitment is restricted to the characteristic prominence were *Pv*UIS4 resides at days 5–7 (Gupta et al., 2019). After day 8, AQP3 starts spreading throughout the PVM and uniform staining of the membrane is observed at 10 dpi (Figures 2C and S2C). The varying spatiotemporal recruitment of AQP3 to hypnozoites verses schizonts highlights the differences between these two liver forms and their need for host AQP3.

### Auphen Treatment Inhibits *P*. *vivax* Liver-Stage Hypnozoites and Schizonts

**Figure 3:**
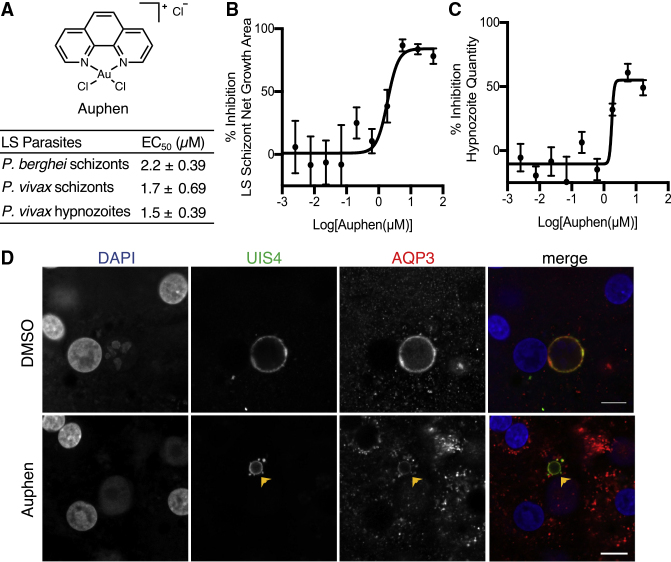
Auphen Inhibits *P*. *vivax* Schizonts and Hypnozoites (A) Table with EC_50_ values for auphen inhibition of *P*. *berghei* and *P*. *vivax* liver stages. Data are shown as average ± SD of three to four independent experiments. (B) Dose-response curve of auphen inhibition of *P*. *vivax* schizont growth area. (C) Dose-response curve of auphen inhibition of *P*. *vivax* hypnozoite quantity per well. Data are shown as the average ± SEM of all independent experiments. (D) Representative confocal images of *P*. *vivax* parasites on day 8 post-infection of PHH in the absence (top panel) or presence of 0.62 μM auphen (bottom panel). Arrow indicates parasite with AQP3 recruitment after auphen treatment. Cells were stained with DAPI (blue), *Pv*UIS4 (green), and *Hs*AQP3 (red). Scale bar, 10 μm.

We previously demonstrated that auphen (Martins et al., 2012), a known *Hs*AQP3 inhibitor, reduces *P*. *berghei* liver-stage schizont size and inhibits *P*. *falciparum* asexual blood-stage parasites (Posfai et al., 2018). Based on previous *in vitro* assays, the compound inhibits glycerol flux but does not influence water or urea permability by the channel up to 100 μM (Martins et al., 2012). To test the effect of auphen on *P*. *vivax* liver forms, we utilized our previously established 384-well HCI assay following a radical cure treatment mode. The treatment begins on 5 dpi, when hypnozoites have matured such that they are no longer susceptible to schizonticidal compounds, such as MMV390048, KDU691, and KAF156, and continues with fresh treatments on days 6 and 7 before fixation on day 8 post-infection (Roth et al., 2018a). We found that auphen inhibits *P*. *vivax* schizont growth area per well and hypnozoite quantity per well with half maximal effective concentration (EC_50_) values of 1.7 ± 0.69 and 1.5 ± 0.39 μM, respectively (Figure 3A). Although liver schizont growth was inhibited by 85%–95% by 8 dpi (Figure 3B), hypnozoite quantity was reduced by 61% when compared with the DMSO control (Figure 3C). A new auphen synthesis was used for these *P*. *vivax* liver-stage assays; therefore, we tested the batch against luciferase-expressing *P*. *berghei* liver schizonts infecting HuH7 hepatocytes. We observed auphen inhibited *P*. *berghei* parasite load with EC_50_ values of 2.2 ± 0.39 μM, similar to a previous report (Posfai et al., 2018). However, unlike previous studies with *P*. *berghei*, we found that *P*. *vivax* schizont quantity per well was reduced at effective doses, indicating that schizont activity was likely cidal and not merely static during treatment (Table S1; Figure S4B).

We further evaluated auphen's effect on liver-stage parasites by performing confocal immunofluorescence microscopy of *P*. *vivax*-infected PHH treated with 0.62 and 1.85 μM auphen. At 0.62 μM auphen, a concentration in which we see some activity against schizonts and hypnozoites, we observed no change in AQP3 localization to the PVM on 8 dpi (Figure 3D). At higher auphen concentrations there were fewer parasites for analysis and observed parasites were difficult to classify as schizonts or hypnozoites. Specifically, at higher doses (1.8–50 μM) a proportion of dying schizonts likely shrink to a size comparable with hypnozoites (<150 μm^2^ area), making the two populations difficult to decipher by HCI. Confocal images of *P*. *vivax*-infected PHH treated with 1.85 μM auphen showed malformed parasites and broken PVMs based on *Pv*UIS4 staining (Figure S4C), suggesting that the parasites may be in the process of dying and clearance. Despite this, AQP3 staining was still observed near the PVM, suggesting that protein recruitment was not affected by the inhibitor.

### AQP3 Plays a Role in *P*. *vivax* Blood-Stage Infection

**Figure 4:**
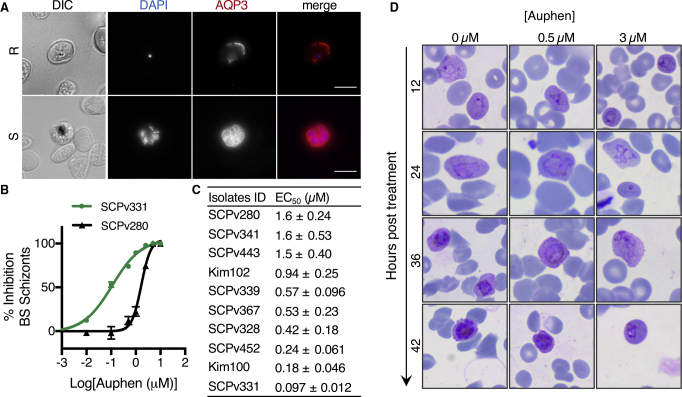
Auphen Inhibits Blood-Stage *P*. *vivax* (A) Representative images of human AQP3 localization in *P*. *vivax*-infected reticulocytes during the ring (top panel) and schizont (bottom panel) stages. Cells were stained with DAPI (blue) and *Hs*AQP3 (red). Scale bar, 10 μm. (B) Representative dose-response curve of auphen inhibition of *P*. *vivax* schizonts in isolates SCPv331 (green circles) and SCPv280 (black triangles). Data are shown as the average ± SEM of all independent experiments. (C) Table of EC_50_ values for auphen inhibition of *P*. *vivax* schizonts in patient isolates. Data are shown as average ± SD of 3 independent experiments. (D) Representative images of *P*. *vivax*-infected clinical isolate in the presence of 0 μM (left column), 0.5 μM (middle column), or 3 μM auphen (right column). Giemsa staining of blood cells revealed a delay in parasite development with auphen treatment.

Inhibition of the acute blood stage of *Plasmodium* infection is necessary to treat malaria. In addition to establishing a role of AQP3 in the *P*. *vivax* liver stage, we sought to determine if the protein is involved in blood-stage infections. Human erythrocytes are naturally abundant in AQP3 (Uhlen et al., 2015) and the protein is recruited to asexual blood-stage *P*. *falciparum* based on *in vitro* studies (Bietz et al., 2009, Posfai et al., 2018). However, *P*. *vivax* asexual blood-stage parasites cannot be maintained in continuous culture. Therefore, clinical isolates collected from Mondulkiri Province, Cambodia, were utilized to test the relevance of AQP3 during the *P*. *vivax* blood stage. After fixing and imaging *P*. *vivax*-infected reticulocytes, we observed AQP3 recruitment to both the ring and schizont forms (Figure 4A). During the ring stage of infection, AQP3 localization was primarily observed around the periphery of the reticulocyte with small puncta associated with the PVM, while the protein appeared to be throughout the parasitophorous vacuole in schizonts. This change in localization may reflect a greater demand for AQP3 as the parasite divides.

Finally, the ability of auphen to inhibit blood-stage *P*. *vivax* development was tested. Clinical isolates at the ring-stage were treated with DMSO or increasing concentrations of auphen throughout the 42–49 h required for ring to schizont development. Auphen inhibited all 10 clinical isolates tested (Figures 4B and 4C) with EC_50_ values ranging from 0.097 to 1.6 μM. The lowest potency measured (SCPv280 = 1.6 ± 0.24 μM) agrees with EC_50_ determinations for auphen activity against the *P*. *vivax* liver stage (Figure 3A). In addition, this potency is similar to auphen's inhibition of the *P*. *falciparum* asexual blood stage (EC_50_ = 0.81 ± 0.10 μM) (Posfai et al., 2018). Unique to these values are isolates with a >10-fold increased auphen sensitivity, such as SCPv331 and Kim100 (0.097 and 0.18 μM, respectively). To further evaluate auphen inhibition, phenotypic evaluation of the parasites was completed throughout inhibitor treatment (0–42 h). We found that auphen treatment (0.5 or 3 μM) inhibited *P*. *vivax* blood-stage development (Figure 4D) instead of exclusively clearing parasites. In general, we observed a greater percentage of parasites in the ring and trophozoite stages after treatment, suggesting that auphen acts primarily as a cytostatic agent against the *P*. *vivax* blood stage.

## Discussion

Molecular interactions that occur between the *Plasmodium* parasite and a hepatocyte remain largely unknown. In particular, the biological processes of liver-resident hypnozoites that are characteristic of *P*. *vivax* are elusive. In this study, we characterize the recruitment of host AQP3 to *P*. *vivax* hypnozoites and liver schizonts as well as asexual blood-stage clinical *P*. *vivax* parasite isolates. During liver-stage infection, AQP3 localizes to the host-pathogen interface in a spatiotemporal manner that differs for actively replicating infections when compared with dormant forms. Host AQP3 recruitment to the PVM is also observed in *P*. *vivax-*infected reticulocytes. Treatment with auphen, a molecule that limits human AQP3 glycerol transport (Martins et al., 2012), inhibits *P*. *vivax* liver- and blood-stage parasites. Our results uncover a role for host AQP3 in the development of *P*. *vivax* liver and blood stages and suggest that modulation of AQP3 activity may be an attractive therapeutic approach.

Within 2–5 days after *P*. *vivax* invasion of PHH, AQP3 is recruited to the PVM. Previously, we have shown that AQP3 gene expression is upregulated after *P*. *berghei* invasion of human hepatocytes and subsequently trafficked to the PVM (Posfai et al., 2018). Host gene expression studies of *P*. *vivax*-infected PHH are technically challenging and hampered by poor signal-to-noise ratios due to the relatively low abundance of parasite-infected hepatocyte RNA versus uninfected hepatocyte RNA. Regardless, our microscopy study suggests an induction of AQP3 protein expression after infection. This induction may be necessary since hepatocytes do not highly express AQP3 (Gregoire et al., 2015). However, the protein is abundant in the cell membranes of erythrocytes, skin, and kidney cells (Ishibashi et al., 1997, Qin et al., 2011, Roudier et al., 1998). We observed AQP3 near the PVM in both the ring and schizont stages of *P*. *vivax*-infected reticulocytes, demonstrating a conserved role of this host protein in various stages of the *Plasmodium* life cycle. This function suggests that the protein may be an attractive early-stage target for parasite load reduction. Consistent with the observation that genetic disruption of AQP3 inhibits *P*. *berghei* liver-stage development (Posfai et al., 2018), we find that auphen inhibits *P*. *vivax* liver and blood stages, suggesting that the recruitment of AQP3 is important for parasite development.

We hypothesize that AQP3 is directed to the host-parasite interface during both the *Plasmodium* liver and blood stages to enable solute transport that assists in parasite development. By 5 dpi, when schizonts can be identified as distinctly larger than hypnozoites, the majority of *P*. *vivax* liver schizonts already have AQP3 recruited to, and dispersed throughout, the PVM. In contrast, hypnozoites have a delayed recruitment of AQP3 and for several days after infection, the protein is at the PVM prominence. As the hypnozoite matures (without nuclear division in its quiescent form), AQP3 disperses throughout the PVM by day 7. The greater abundance of the protein during the multi-nucleated liver and blood stages suggests that the solute(s) transported by the protein are more critical at this time when compared with early stages of development. Indeed, the swift recruitment of AQP3 at a time that coincides with *P*. *vivax* replication and expansion hints that the protein may be important for this process. This contrasts with the relatively delayed recruitment in non-replicating hypnozoites. Human AQP3 is known to flux water, glycerol, urea, and hydrogen peroxide (Echevarria et al., 1994, Ishibashi et al., 1994, Zhao et al., 2006). Thus, if the selectivity filter of AQP3 is unmodified by *Plasmodium* it could be transporting a number of small molecules throughout infection into or out of the host cytosol. The parasite also encodes a single aquaglyceroporin that localizes to the parasite's plasma membrane where it aids in *P*. *berghei* development within hepatocytes (Promeneur et al., 2007, Promeneur et al., 2018). Liver-stage parasites use their aquaglyceroporin to obtain exogenous glycerol for glycerophospholipid generation, an important component of cell membranes. The parasite aquaglyceroporin does not localize to the PVM; therefore, the host AQP3 is presumably needed to acquire molecules from the host cytosol. The proposal that both liver forms require solute flux is further supported by transcriptomic data. RNA sequencing studies from *P*. *cynomolgi* (Bertschi et al., 2018) and *P*. *vivax* (Gural et al., 2018) show that the parasite aquaglyceroporin is expressed in liver schizonts and hypnozoites, although at a lower abundance during dormancy. Thus, while dormant, hypnozoites express pathways for energy metabolism, and the host AQP3 could support these processes through solute flux. Alternatively, AQP3 could provide a mechanism by which the parasite dispenses toxins, such as urea, the byproduct of protein metabolism, or hydrogen peroxide. Unfortunately, due to limitations in acquiring large numbers of *P*. *vivax*-infected hepatocytes, our inability to isolate parasites from within hepatocytes, and insufficient mass spectrometry sensitivity, it is not yet possible to resolve molecules that AQP3 transports to or from the parasite to facilitate survival.

Currently, primaquine and tafenoquine are the only antimalarials approved to clear *P*. *vivax* hypnozoites for prevention of recurring malaria (John et al., 2012, Lacerda et al., 2019). Here, we found that auphen inhibits *P*. *vivax* liver schizonts and hypnozoites (EC_50_ = 1.7 and 1.5 μM, respectively) with a similar potency as the compound's activity against *P*. *berghei* liver schizonts (EC_50_ = 2.2 μM). Auphen is a known AQP3 inhibitor, but we were unable to validate its mechanism of action in our system due to technical challenges associated with infecting cells with AQP3 disruption (low parasite signal) or overexpression (misfolded protein). Although auphen's target in *Plasmodium*-infected cells awaits to be experimentally verified, we found it reduced liver-stage schizont size without altering the recruitment of AQP3 to the PVM. A similar liver schizont growth inhibition phenotype has been described for several antimalarials, including antibiotics (Mahmoudi et al., 2003) and KAF156 (Gural et al., 2018); however, in this study we were not able to directly assess the mechanism of parasite death or clearance from PHHs. For hypnozoite activity, the rate of clearance is of note as we found auphen treatment causes a 61% reduction in hypnozoites per well only 72 h after the first application to 5 dpi hypnozoites. Conversely, hypnozoites treated with primaquine hours after invading a host hepatocyte, when the hypnozoite is still sensitive to many prophylactic compounds, take weeks to clear from *in vitro* culture; and successful primaquine treatment of fully quiescent *P*. *vivax* hypnozoites, as defined by insensitivity to PI4K inhibitors after around 5 dpi (Figure S4), has yet to be demonstrated *in vitro* (Gural et al., 2018, Zeeman et al., 2016). Tafenoquine was developed as a more potent alternative to primaquine and was recently shown to have an effect on 5 dpi hypnozoites of *P*. *cynomolgi*, a zoonotic sister species of *P*. *vivax*, in a primary hepatocyte-based *in vitro* assay following a similar dosing scheme (4–7 dpi) and endpoint (8 dpi) as we performed herein (Gupta et al., 2019). Similar to our results with auphen-treated *P*. *vivax* hypnozoites, 70% of tafenoquine-treated *P*. *cynomolgi* hypnozoites were cleared from culture after only 72 h of treatment, suggesting a more rapid, and possibly shared, clearance mechanism. Although we can only speculate as to what hepatic processes contribute to clearance, a delicate balance of lysosome recruitment to the PVM of intrahepatic *Plasmodium* parasites has been described, and, given that AQP3 is recruited to the PVM, it is possible that auphen treatment directly or indirectly alters lysosome-PVM fusion dynamics and could lead to autophagy of liver forms (Boonhok et al., 2016, Niklaus et al., 2019). However, such a mechanism would not explain a clearance mechanism for auphen activity against blood-stage parasites, thus treatment could cause parasite death by deprivation or buildup of the unidentified solute(s) traversing AQP3, and for liver forms, an unhealthy parasite's inability to balance lysosome-PVM fusion could lead to rapid clearance. These hypotheses will require detailed molecular studies to resolve.

Auphen also inhibited the *P*. *vivax* asexual blood stage where a delay in schizont development was observed upon treatment. Interestingly, a range of EC_50_ values were observed among the 10 isolates tested. The strains with the highest EC_50_ values (EC_50_ = 1.6–0.94 μM) matched the activity expected based on our liver-stage *P*. *vivax* and *P*. *berghei* dose-response analysis as well as the potency observed against the *P*. *falciparum* 3D7 asexual blood stage. Intriguingly, some isolates were hypersensitive to the compound, where as high as a 16-fold increase in potency was observed (EC_50_ = 0.097 μM). One explanation for the varying potencies could be a natural variation of AQP3 within a population. Population genetic studies have identified allelic differences, including AQP3 null individuals (Roudier et al., 2002). Thus, it is possible that these host variations affect auphen potency or alternatively differences exist in the parasite strains. If AQP3 dependency varies among *P*. *vivax* isolates it would affect the sensitivity of drugs targeting this protein. Although the molecular basis for the observed variation in auphen potency remains to be determined, the increased drug susceptibility observed encourages future studies in the context of genetic variation within the *P*. *vivax* and host genomes. Such a study would also resolve the molecular target(s) of auphen responsible for parasite inhibition. The compound is known to inhibit glycerol flux by human AQP3, but it may inhibit other host or parasite processes to reduce *Plasmodium* parasite burden.

Herein, we demonstrate a role for human AQP3 in various stages of the *P*. *vivax* life cycle. The recruitment of this host protein to the PVM in both mouse- and human-infective species of *Plasmodium*, in addition to multiple life-cycle stages, suggests a conserved role for AQP3 at the host-parasite interface. From a drug discovery and development perspective, it has been proposed that the next generation of radical cure antimalarials should include up to four different chemical profiles with activity against several life-cycle stages, including liver schizonts, hypnozoites, blood schizonts, and gametocytes (Burrows et al., 2017). Our finding that auphen inhibits *P*. *vivax* hypnozoites as well as liver and blood schizont development demonstrates the importance of its molecular target. Future work will resolve the function of AQP3 throughout infection to understand the molecules it filters to assist in parasite development, and, in doing so, could unveil additional molecular targets from which target-based radical cure drug discovery could progress. With few compounds known with activity against the dormant parasite, this discovery advances our understanding of biological processes that occur during this stage as well adds to our repertoire of anti-hypnozoite targets.

## Significance

**Much is unknown about *P*. *vivax* liver-stage biology despite its global prevalence. When *P*. *vivax* parasites invade a hepatocyte, they form either a liver schizont or hypnozoite. Schizonts contain thousands of nucleated parasites capable of causing clinical malaria after erythrocyte invasion. Alternatively, hypnozoites can remain dormant for days or weeks before activating to cause disease. Using a recently developed *P*. *vivax* liver-stage model system we examined the role of host AQP3 in hypnozoite and schizont development within hepatocytes as well as the role of the protein in the *P*. *vivax* blood stage. We observed the host AQP3 is recruited to the host-parasite interface after infection is established. The spatiotemporal pattern of this recruitment varies for hypnozoites and schizonts, highlighting differences in its role for the two liver-stage parasite forms. Localization of human AQP3 is also observed *ex vivo* in *P*. *vivax* blood isolates. Importantly, a known AQP3 inhibitor has anti-hypnozoite activity and reduces *P*. *vivax* liver-stage and blood-stage burden. Our findings highlight the critical role of host AQP3 in *Plasmodium* infection and suggest its function may be modulated to facilitate disease control efforts.**

## STAR★Methods

### Key Resources Table

**Table:** 

REAGENT or RESOURCE	SOURCE	IDENTIFIER
**Antibodies**		

recombinant mouse monoclonal antibody against PvUIS4	Noah Sather Laboratory, Center for Infectious Disease Research, Seattle, WA USA; (Schafer et al., 2018)	rPvUIS4
mouse monoclonal antibody against Plasmodium EXP2	The European Malaria Reagent Repository	Cat# 7.7 (Anti-EXP-2)
IgG (H+L) Cross-Adsorbed Goat anti-Mouse, Alexa Fluor® 488, Invitrogen™	Thermo Fisher Scientiftic	Cat# A11001; RRID: AB_2534069
rabbit antibody against human Aquaporin 3	Rockland antibodies	Cat# 600-401-E91
IgG (H+L) Cross-Adsorbed Donkey anti-Rabbit, Alexa Fluor® 568, Invitrogen™	Thermo Fischer Scientific	Cat# A10042; RRID: AB_2534017

**Biological Samples**		

*Plasmodium vivax* Patient Blood Sample	Institut Pasteur du Cambodge	SCPv756
*Plasmodium vivax* Patient Blood Sample	Institut Pasteur du Cambodge	SCPv700
*Plasmodium vivax* Patient Blood Sample	Institut Pasteur du Cambodge	SCPv629
*Plasmodium vivax* Patient Blood Sample	Institut Pasteur du Cambodge	SCPv611
*Plasmodium vivax* Patient Blood Sample	Institut Pasteur du Cambodge	SCPv280
*Plasmodium vivax* Patient Blood Sample	Institut Pasteur du Cambodge	SCPv341
*Plasmodium vivax* Patient Blood Sample	Institut Pasteur du Cambodge	SCPv443
*Plasmodium vivax* Patient Blood Sample	Institut Pasteur du Cambodge	Kim102
*Plasmodium vivax* Patient Blood Sample	Institut Pasteur du Cambodge	SCPv339
*Plasmodium vivax* Patient Blood Sample	Institut Pasteur du Cambodge	SCPv367
*Plasmodium vivax* Patient Blood Sample	Institut Pasteur du Cambodge	SCPv328
*Plasmodium vivax* Patient Blood Sample	Institut Pasteur du Cambodge	SCPv452
*Plasmodium vivax* Patient Blood Sample	Institut Pasteur du Cambodge	Kim100
*Plasmodium vivax* Patient Blood Sample	Institut Pasteur du Cambodge	SCPv331
Pooled AB Human Serum	The Interstate Blood Bank, Inc	N/A
Human Serum	Cambodia National Blood Transfusion Center	N/A

**Chemicals, Peptides, and Recombinant Proteins**		

Auphen	Derbyshire Lab, Duke University, (Posfai et al., 2018)	CAS# 14910-99-7
Monensin Sodium Salt	MilliporeSigma	CAS# 22373-78-0; Cat# M5273-500mg; Lot# SLBK4090V
Nigericin Sodium Salt	MilliporeSigma	CAS# 28643-88-3; Cat# N7143-5mg; Lot# 026M4144V
MMV390048	Specs	CAS# 1314883-11-8, Lot# MMV390048-09

**Critical Commercial Assays**		

Bright-Glo Luciferase Assay System	Promega	Cat# E2610
CellTiter-Fluor Cell Viability Assay	Promega	Cat# G6080

**Experimental Models: Cell Lines**		

HuH7 hepatocytes	Dr. Peter Sorger, Harvard Medical School	RRID: CVCL_0336
Cryopreserved Male Primary Human Hepatocytes, Lot BGW	BioIVT	Cat# M00995-P
Cryopreserved Male Primary Human Hepatocytes, Lot UBV	BioIVT	Cat# M00995-P

**Experimental Models: Organisms/Strains**		

*Anopheles stephensi* mosquitoes	NYU Langone Medical Center Insectary Core Facility	RRID: SCR_012350
*P*. *berghei* luciferase-expressing sporozoites	NYU Langone Medical Center Insectary Core Facility (Franke-Fayard et al., 2005)	RRID: SCR_012350
*Anopheles dirus* mosquitoes, A strain	Institut Pasteur du Cambodge; Siv Sovannaroth, National Center for Parasitology, Entomology and Malaria Control of Cambodia (St. Laurent et al., 2015)	NA

**Oligonucleotides**		

Nested real-time PCR Pf_forward	Merck-Sigma Custom Oligos	ATGGATATCTGGATTGATTTTATTTATGA
Nested real-time PCR Pf_reverse	Merck-Sigma Custom Oligos	TCCTCCACATATCCAAATTACTGC
Nested real-time PCR Pv_forward	Merck-Sigma Custom Oligos	TGCTACAGGTGCATCTCTTGTATTC
Nested real-time PCR Pv_reverse	Merck-Sigma Custom Oligos	ATTTGTCCCCAAGGTAAAACG
Nested real-time PCR Pm_forward	Merck-Sigma Custom Oligos	ACAGGTGCATCACTTGTATTTTTTC
Nested real-time PCR Pm_reverse	Merck-Sigma Custom Oligos	TGCTGGAATTGAAGATAATAAATTAGTAATAACT
Nested real-time PCR Po_forward	Merck-Sigma Custom Oligos	GTTATATGGTTATGTGGAGGATATACTGTT
Nested real-time PCR Po_reverse	Merck-Sigma Custom Oligos	CGAATGGAAGAATAAAATGTAGTACG
Primary PCR Forward	Merck-Sigma Custom Oligos	TGGAGTGGATGGTGTTTTAGA
Primary PCR Reverse	Merck-Sigma Custom Oligos	ACCCTAAAGGATTTGTGCTACC

**Software and Algorithms**		

Prism version 7	GraphPad Software	RRID: SCR_002798; graphpad.com
CDD Vault	CDD Vault	collaborativedrug.com
MetaXpress	Molecular Devices	RRID: SCR_016654
Gen5 version 3.05	Biotek	N/A
Imaris 9.0	Oxford instruments	RRID: SCR_007370
Fiji	ImageJ	RRID: SCR_002285; https://imagej.net/Fiji/Downloads

**Other**		

40nL Pin Tool	V&P Scientific	N/A
ImageXpress Confocal Micro	Molecular Devices	N/A
Lionheart FX	Biotek	N/A
880 AiryScan Inverted Confocal	Zeiss	N/A
Envision Plate Reader	Perkin Elmer	N/A
Leica DM2500	Leica Microsystem	N/A

### Lead Contact and Materials Availability

Further information and requests for *P*. *vivax-*related reagents should be directed to Benoît Witkowski (bwitkowski@pasteur-kh.org). There are restrictions to the availability of some *P*. vivax-related reagents due to inadequate methodology for preservation and propagation of the parasites in clinical isolates. This study did not generate new unique reagents from Emily Derbyshire. Further information about auphen and imaging reagents should be directed to and will be fulfilled by the Lead Contact, Emily Derbyshire (emily.derbyshire@duke.edu).

### Experimental Model and Subject Details

#### Study Sites & Clinical Isolate Collection

Blood samples were collected from symptomatic *P*. *vivax* patients at local health facilities in Modulkiri & Rattanakiri provinces (eastern Cambodia) from 2018-2019. Clinical isolate collection and research procedures were reviewed and approved by the Cambodian National Ethics Committee for Health Research (approval number: #101NECHR, #270NECHR & #273NECHR). Patients presenting signs of severe malaria, infected with non-vivax malaria parasites, under 5 years of age, or who were pregnant or lactating were excluded form the collection. Following informed consent from eligible study participants, venous blood samples were collected by venipuncture into heparin-containing tubes (Beckton Dickinson, Cat# 367886). Immediately after collection, an artesunate-mefloquine course was provided according to Cambodia National Malaria Treatment Guidelines. Clinical isolates intended for blood stage culture were leukocyte-depleted by NWF filtration (ZXbio, China), cryopreserved in glycerlolite-57 (Fresenius Kabi, Cat# FWL4A7831) and stored in liquid nitrogen prior to use. Clinical isolates for sporozoite production were immediately prepared for feeding to *An*. *dirus* mosquitoes in a secure insectary in Mondulkiri Province, Cambodia. The *An*. *dirus* *A* colony was isolated from Veal Veng District, Pursat Province, Western Cambodia, in July 2011 (St. Laurent et al., 2015). This colony is maintained by the National Center for Parasitology, Entomology and Malaria Control of Cambodia. Eggs were provided to the Institute Pasteur of Cambodia under MTA. Collected blood was pelleted by centrifugation at 37°C for 5 min at 3000 rpm. Serum was replaced with the same volume of heat-inactivated naive human AB serum (Interstate Blood Bank). The serum-blood mixture was maintained at 37°C using a custom-made water-jacketed glass insect feeding bell and 5-7-day old adult female mosquitoes were allowed to engorge for 1 hr. *Plasmodium* species typing was confirmed by a previously-described 2-step semiquantitative RT-PCR on genomic DNA isolated from cryopreserved blood samples (Canier et al., 2013). The primary polymerase chain reaction amplification was performed in a 20 μl reaction mixture containing 5 μL of DNA template, 1.25 U Hot FirePol DNA Polymerase (Solis BioDyne, Cat# 01-02-00500), 200 μM dNTP mix (Solis BioDyne, Cat# 02-31-00020), and 250 nM of the forward and reverse primers (see Key Resource Table for oligo sequences). The primary PCR was performed with an initial activation step at 95°C for 15 min, followed by 20 cycles consisting of a denaturation step at 94°C for 30 seconds, an annealing step at 58°C for 1 min, an extension step at 72°C for 1 minute 30 seconds, and a final extension step at 72°C for 10 minutes. Following the primary PCR, a nested real-time polymerase chain reaction amplification was performed in 20 μl of reaction mixture containing 5 μl of template Primary PCR product diluted 1:10 in water, 1x Hot FirePol Evagreen HRM Mix (Solis Biodyne, Cat# 08-24-00001) and 250 nM of the forward and reverse primers for one of each of the four *Plasmodium* species (see Key Resource Table for oligo sequences); with one reaction mixture per species-specific primer set. The nested PCR was performed with an initial activation step at 95°C for 15 min, followed by 40 cycles consisting of a denaturation step at 95°C for 10 seconds, an annealing step at 62°C for 20 seconds, and an extension step at 72°C for 20 seconds.

#### Primary Human Hepatocyte Culture

Vials of cryopreserved PHH (BioIVT) were shipped to IPC and thawed into InVitroGro™ CP Medium (BioIVT, Cat# Z99029) containing a 1x antibiotic mixture (PSN, Gibco Cat# 15640055 and Gentamicin, Gibco, Cat# 15710072). Cell viability was recorded using trypan blue exclusion on a hemocytometer, and 18,000 live cells were added to each well of a collagen-coated 384-well plate (Grenier, Cat# 781956). Cultures were maintained in a standard tissue culture incubator at 37°C and 5% CO_2_. Media was changed thrice weekly with CP media with antibiotics. Two lots of PHH were used to determine auphen activity and AQP3 localization. Lot BGW were obtained from a 50-year old Caucasian male, lot UBV were obtained from a 57-year old Caucasian male.

#### Obtaining *P. berghei* Sporozoites and Maintaining HuH7 Cell Cultures

HuH7 hepatocytes were obtained from Dr. Peter Sorger (Harvard Medical School) and maintained in Dulbecco’s Modified Eagle Medium (DMEM) with L-glutamine (Gibco) supplemented with 10% (v/v) heat-inactivated fetal bovine serum (Sigma-Aldrich) and 1% (v/v) antibiotic-antimycotic (Thermo Fisher Scientific) in a standard tissue culture incubator (37°C, 5% CO_2_). *A*. *stephensi* mosquitoes infected with luciferase-expressing *P*. *berghei* (Franke-Fayard et al., 2005) were obtained from NYU Langone Medical Center Core Facility. Mosquitoes were kept at room temperature in an incubator with a water bath to maintain humidity and fed 10% sucrose daily until dissections to obtain sporozoites from the salivary galnds.

### Method Details

#### Collection of *P. vivax* Sporozoites, Liver Stage Infection, Drug Treatment, and HCI

*P*. *vivax* infections were completed as previously described (Roth et al., 2018a). In summary, following a *P*. *vivax* gametocyte-containing bloodmeal, *An*. *dirus* mosquitoes were maintained on a natural light cycle and 10% sucrose in water. Mosquitoes found positive for *P*. *vivax* oocysts at six-days post feeding were transported to the IPC facility in Phnom Penh, Cambodia where salivary glands were aseptically dissected into RPMI without sodium bicarbonate (Gibco, Cat# 61870-010) on day 16-21 post feeding. PHH (BioIVT) were seeded as described above 2-3 days prior to infection with 5,000-20,000 sporozoites per well. Infection was performed by diluting freshly dissected sporozoites into CP media with antibiotics, adding 20 μL sporozoite-media mixture to each well, and centrifugation of the 384-well plate at 200 RCF for 5 min at room temperature. Media was exchanged with fresh CP media containing antibiotics the day after infection and every 2-3 days thereafter. For dose-response assays, powdered compounds were diluted in sterile DMSO (Tocris) and plated in a semilog (1:3) dose response from 50 mM to 280 nM (for auphen) or 3.3 mM to 20 nM (for monensin and MMV390048 controls) in a low-volume, sterile 384-well plate (Grenier, Cat# 784261). Infected PHHs were treated on 5, 6, and 7 days post infection (dpi) using either a custom-manufactured pin tool (V&P Scientific) designed to transfer 40 nL from a source plate containing compounds diluted in DMSO to a destination plate containing *P*. *vivax*-infected PHHs in 40 μL of CP media, thus achieving a 1,000-fold dilution of compound upon treatment, or by dilution of compound into plated media by hand pipetting. Media was exchanged with fresh CP media with antibiotics immediately before daily compound treatment. At 8 dpi cultures were fixed for 1 hour with 4% paraformaldehyde (ThermoFisher Scientific) in PBS (Gibco). Fixed cultures were stained with recombinant mouse anti-*P*. *vivax* Upregulated in Infectious Sporozoites-4 antibody (rPvUIS4, (Schafer et al., 2018) ) diluted 25,000-fold in staining buffer (0.03% TritonX-100, 1% (w/v) BSA in PBS) overnight at 4°C. Stained cultures were washed thrice with PBS and then stained with rabbit anti-mouse Alexfluor488-conjugated antibody (ThermoFisher Scientific, Cat# A11001) diluted 1:1000 in staining buffer overnight at 4°C. Cultures were then washed thrice with PBS and counterstained with 1 μg/mL Hoechst 33342 (ThermoFisher Scientific, Cat# H3570) to detect parasite and host cell nuclear DNA. Cultures were washed once more and stored in PBS prior to automated High Content Imaging with a 20x objective on a ImageXpress Confocal Micro (Molecular Devices) or 4x objective on a Lionheart (Biotek). Liver stage parasites were quantified for number and growth area per well using provided cellular analysis and quantification software (MetaXpress for ImageXpress or Gen5 for Lionheart). Hypnozoites were defined as brightly-UIS4-stained round forms (ratio of maximum and minimum widths of each form > 0.6) with under 150-180 μm^2^ total area and a bright prominence in the PVM. Schizonts were defined as brightly-UIS4-stained forms with greater than 500 μm^2^ total area. HCI analysis parameters were slightly tailored for each run using MMV390048-treated hypnozoites to help determine hypnozoite stain intensity and size cutoffs.

#### *P. berghei* Liver Stage Infections

To test the effects of auphen, HuH7 cells were treated with auphen and infected with *P*. *berghei* ANKA sporozoites. To infect hepatocytes, 7x10^3^ HuH7 cells were seeded in each well of a 384-well plate (Corning, Cat# 3570) and maintained at 37°C, 5% CO^2^. The following day, luciferase-expressing *P*. *berghei* sporozoites were freshly dissected from the salivary glands of *A*. *stephensi* mosquitoes and quantified microscopically. Immediately prior to infection, hepatocytes were treated with 20–0.01 μM auphen or a DMSO control. DMSO concentration was normalized in all wells to 1%. The monolayer of HuH7 cells was infected with 4x10^3^ sporozoites and the plate was centrifuged at 800 RCF for 10 minutes. HuH7 viability and *P*. *berghei* parasite load of infected hepatocytes was measured 48 hpi by adding CellTiter-Fluor and Bright-Glo (Promega, Cat# G6080 and Cat# E2610) reagents, respectively. Fluorescence and luminescence signals were measured with an EnVision plate reader (Perkin Elmer).

#### *P. vivax* Blood Stage Culture & Phenotyping

*In vitro* susceptibility to auphen was assessed using schizont maturation assays. Cryopreserved *P*. *vivax*-infected clinical isolates (described above) were thawed by dropwise addition of decreasing concentration of NaCl solution in water (12%, 1.2% & 0.9%). Short term *in vitro* culture was performed at 37°C under 5% O2 and 5% CO2 in IMDM medium (Gibco, Cat# 12440079) supplemented with 2.5% human serum (Cambodia National Blood Transfusion Center) and 0.5% Albumax II (Gibco, Cat# 11021029). Samples showing less than 3000 parasites per μl of blood or less than 75% of ring stages were excluded. Parasites were treated with 0.1% DMSO (positive growth control) or a concentration gradient of auphen in 0.1% DMSO (7 points, from 10 nM to 10 μM). The assay time for schizont maturation was between 42 to 49 hrs following initial parasite staging. Experiments were stopped when at least 40% of the parasites in positive controls had reached a four-nuclei schizont stage. The number of schizonts verses all parasites was scored by quantification of Giemsa-stained (Merck, Cat# 109204) thick blood smears analyzed by light microscopy and counts were used to generate a dose-response curve and EC_50_ value. Measurements were repeated three times for each isolate. Analysis of parasite morphology upon auphen exposure was performed by first thawing parasites using the same culture conditions as above followed by Percoll (Sigma-Aldrich, Cat#P1644) enrichement (Rangel et al., 2018). Parasite micrographs were obtained from 5% Giemsa-stained thin blood smears imaged with a DM2500 widefield fluorescent microscope (Leica Microsystems).

#### Localization of Aquaporin-3 and EXP2

PHH were infected with *P*. *vivax* sporozoites and individual plates were fixed between days 1-10 dpi as described above. Fixed cultures were washed thrice with PBS and blocked with 3% BSA for 30 min at room temperature. Cultures were simultaneously incubated with rabbit anti-*Hs*AQP3 (Rockland, Cat# 600-401-E91) diluted 100-fold and rPvUIS4 (Schafer et al., 2018) diluted 10,000-fold in stain buffer overnight at 4°C. Cultures were then washed thrice with PBS and incubated in donkey anti-rabbit AlexaFluor 568 (ThermoFisher Scientific, Cat# A10042) diluted 400-fold and goat anti-mouse AlexaFluor 488 (ThermoFisher Scientific, Cat# A11001) diluted 5000-fold for 1 hr at room temperature. Cultures were washed thrice with PBS and incubated with 0.5 μg/mL DAPI (ThermoFisher Scientific, Cat# D1306) for 7 min. Cells were washed once in PBS before imaging. Images were taken using an 880 Airyscan inverted confocal microscope (Zeiss) and analysis was done with ImageJ (Fiji). Percent localization of AQP3 to *Pv*UIS4 was analyzed through area using ImageJ (Schindelin et al., 2012). Three-dimensional images were constructed using Imaris software (Bitplane). For time course studies, 3-5 wells were fixed and analyzed for each day.

Percoll enriched infected reticulocytes from a cryopreserved clinical isolate (SCPv443) were fixed 42 hrs post thaw, permeabilized and blocked as described previously. Incubations with anti-*Hs*AQP3 primary antibody and secondary antibody were completed as described above for liver stage infections. After nuclear staining with 1 μg/mL Hoechst 33342, cells were imaged on a DM2500 widefield fluorescent microscope.

To analyze EXP2, *P*. *vivax*-infected PHHs were fixed eight days post infection. Infected wells were then permeabilized for 20 minutes with 0.2% TritonX-100, washed thrice with PBS, and blocked in 3% BSA for one hour. Primary antibodies rabbit anti-*Hs*AQP3 and mouse anti-*Pv*EXP2 (The European Malaria Reagent Repository, Cat# 7.7) were sequentially added, diluted 1:100 and 1:500, respectively. Antibodies were incubated for 48 hrs at 4°C and washed 3 times with PBS before addition of 1:400 AlexaFluor 568 donkey anti-rabbit for *Hs*AQP3 or 1:400 AlexaFluor 488 goat anti-mouse for *Pv*EXP2. Secondary antibodies were incubated 1.5 hours at room temperature. Cells were washed thrice with PBS before addition of 0.5 μg/mL DAPI for 10 minutes. Cells were washed 3 final times with PBS before image collection with an 880 Airyscan inverted confocal microscope. Image analysis was completed using ImageJ.

### Quantification and Statistical Analysis

An auphen dose response against *P*. *vivax* liver forms was obtained from four independent experiments, in which a different *P*. *vivax* clinical isolate was allowed to infect UBV or BGW human hepatocytes. All four independent experiments resulted in sufficient hypnozoite formation within each well for dose response calculations, however, for one isolate fewer schizonts formed per well and the schizont data were not used (Table S1). Liver stage parasite growth metrics and compound dilutions were loaded into CDD Vault (Collaborative Drug Discovery) and growth data normalized such that zero (negative) inhibition was set as the average of DMSO wells and 100% (positive) inhibition was set to the effective doses of a Monensin control (Roth et al., 2018a). Percent inhibition for each set of replicate wells was calculated using:

$$\%\;Inhibition=\;100\;\times \left(\frac{Raw\;Data\;-\;Average\;Negative\;Control}{Average\;Positive\;Control\;-\;Average\;Negative\;Control}\right)\;$$

Compounds activity was determined by a dose-response curve fit using the Levenberg–Marquardt algorithm (Levenberg, 1944, Marquardt, 1963) to calculate EC_50_s and maximal inhibition. Outliers were removed within the user interface of CDD Vault. Of note, the highest dose of auphen (50 mM concentration in the source drug plate, or 50 μM concentration in the assay plate after pin tool transfer) was poorly soluble and resulted in fluorescent, crystalized compound in the well. As most compounds are assayed in our *P*. *vivax* liver stage platform from 50 μM to reduce the risk of false negative activity due to metabolic liability, such high doses often result in similar solubility issues, resulting in confounded HCI data. Thus, the data from the highest dose were marked as outliers and not used in curve fit calculations. MMV390048 was also included as a reference compound for inhibition of *P*. *vivax* schizonts. The reported EC_50_ values are the average +/- the standard error of the mean. Individual biological replicates of auphen activity against *P*. *vivax* blood stages are listed in Figure 4C. Graphpad (Prism) was used to create publication-quality charts (Figures 3, 4, S3, and S4).

### Data and Code Availability

This study did not generate datasets.

## Supplemental Information

Document S1. Figures S1–S4

Table S1. Raw *P. vivax* Liver-Stage Growth Data in Response to Auphen Treatment, Related to Figure 3 The number of hypnozoites and schizonts, as well as the net growth area of schizonts per well (in μm^2^) are shown for all independent replicates performed.

Document S2. Article plus Supplemental Information
