# Supplementary material for: Plasmodium vivax Liver and Blood Stages Recruit the Druggable Host Membrane Channel Aquaporin-3
Source: Cell Chem Biol. 2020 Jun 18;27(6):719–727.e5. doi: 10.1016/j.chembiol.2020.03.009 (PMC7303948; doi:10.1016/j.chembiol.2020.03.009)
Supplement: Document S1. Figures S1–S4 [file mmc1.pdf]

Cell Chemical Biology, Volume *27*

Supplemental Information

Plasmodium vivax Liver and Blood Stages Recruit the Druggable Host Membrane Channel Aquaporin-3

Dora Posfai, Steven P. Maher, Camille Roesch, Amélie Vantaux, Kayla Sylvester, Julie Péneau, Jean Popovici, Dennis E. Kyle, Benoît Witkowski, and Emily R. Derbyshire

Supplemental Information

Plasmodium vivax liver and blood stages recruit the druggable host membrane channel aquaporin-3

Dora Posfai*, Steven P. Maher*, Camille Roesch, Amélie Vantaux, Kayla Sylvester, Julie Péneau, Jean Popovici, Dennis E. Kyle, Benoît Witkowski†, and Emily R. Derbyshire†

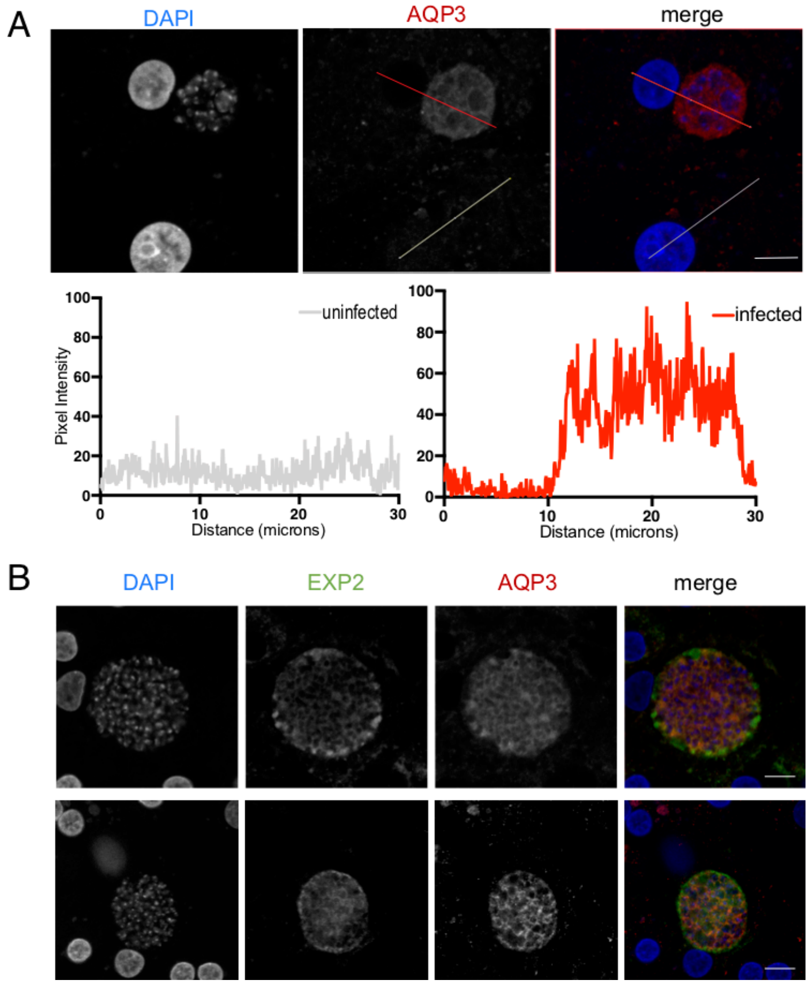

Figure S1, related to Figure 1. AQP3 localization during *P. vivax* infection of PHH. A) PHH infected with *P. vivax* are stained 8 dpi with anti-*Hs*AQP3 (red) and DAPI (blue). Lines indicate measurement for pixel intensity in uninfected (grey) and infected (red) cells. Quantification shown below images is pixel intensity vs distance across each line. B) *P. vivax*-infected PHH stained with anti-*Pv*EXP2 (green), anti-*Hs*AQP3 (red), and DAPI (blue) on 8 dpi. Two represented cells are shown. Merged images show that *Hs*AQP3 staining does not completely overlap with *Pv*EXP2 staining. Scale bar = 10 μM.

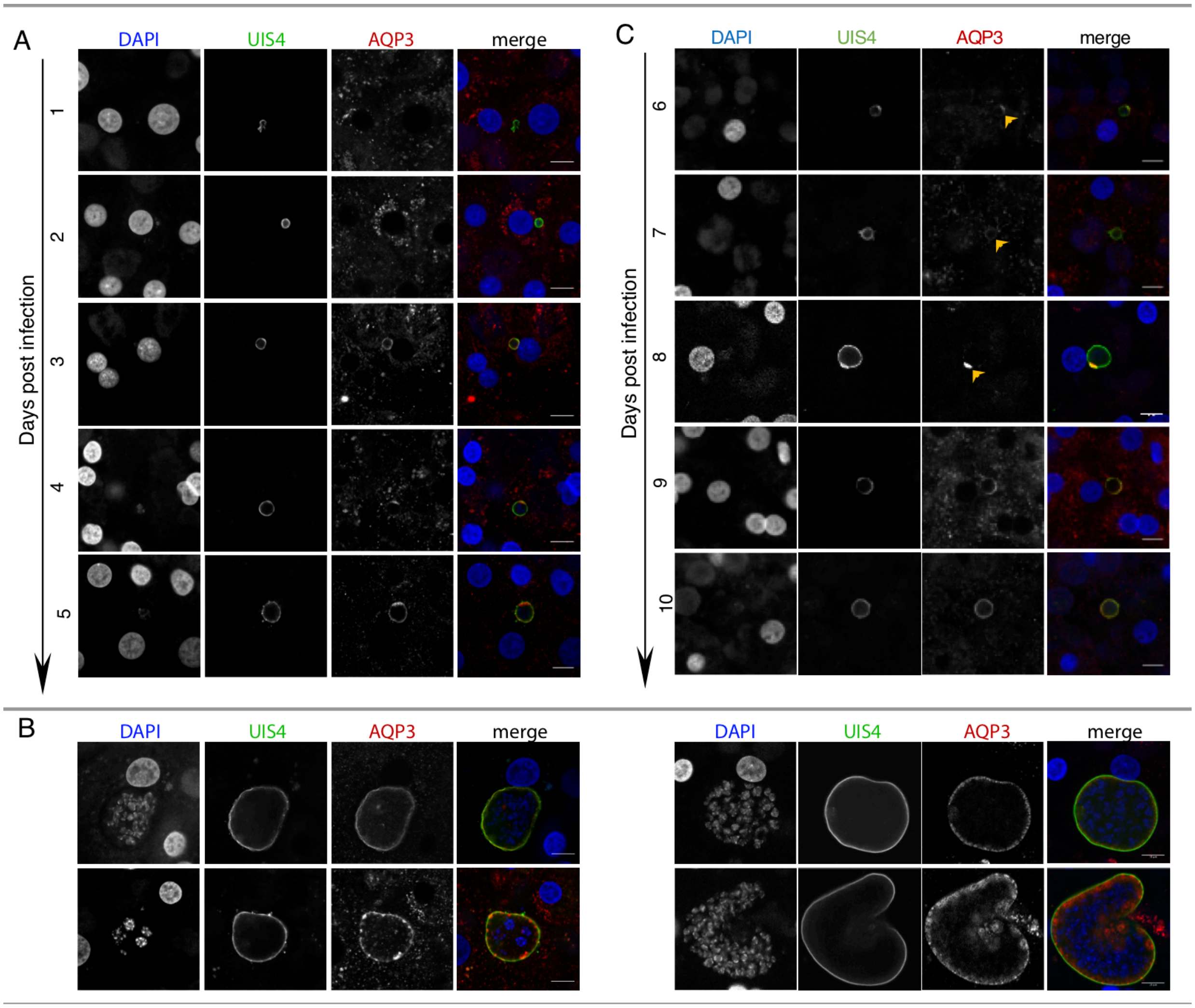

Figure S2, related to Figure 2. AQP3 localization to parasitophorous vacuole during *P. vivax* liver-stage schizont development. A) Images of primary hepatocytes infected with *P. vivax* from day 1 through 5 prior to distinction between forms. B) Large mature schizont morphologies of various cells showing variability of infection and *Hs*AQP3 patterns (red). C) PI4K inhibitor (MMV390048)-treated cells infected with *P. vivax*, showing hypnozoites from day 6-10. Arrows indicate *Hs*AQP3 localization to hypnozoites. Cells stained with *Pv*UIS4 (green), *Hs*AQP3 (red), and DAPI (blue). Scale bar = 10 μM.

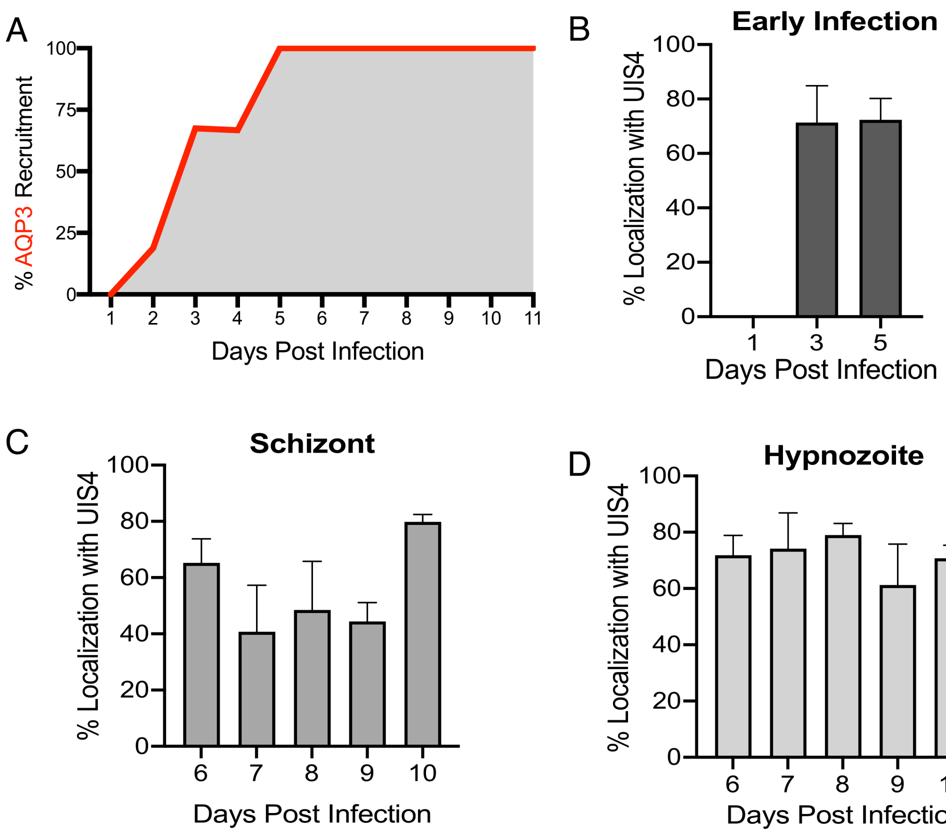

Figure S3, related to Figure 2. AQP3 recruitment and UIS4 co-localization in *P. vivax* liver-stage parasites. A) Percent of cells with AQP3 localization was determined throughout infection (days 1-11). Every *P. vivax*-infected cell displayed AQP3 recruitment by day 5. Population quantified was a mixture of small and large liver-stage forms. Percent of AQP3 colocalizing with *Pv*UIS4 staining in B) days 1-5 early infections and days 6-11 C) schizonts and D) hypnozoites. Data shown is average colocalization ± SEM with at least two infections.

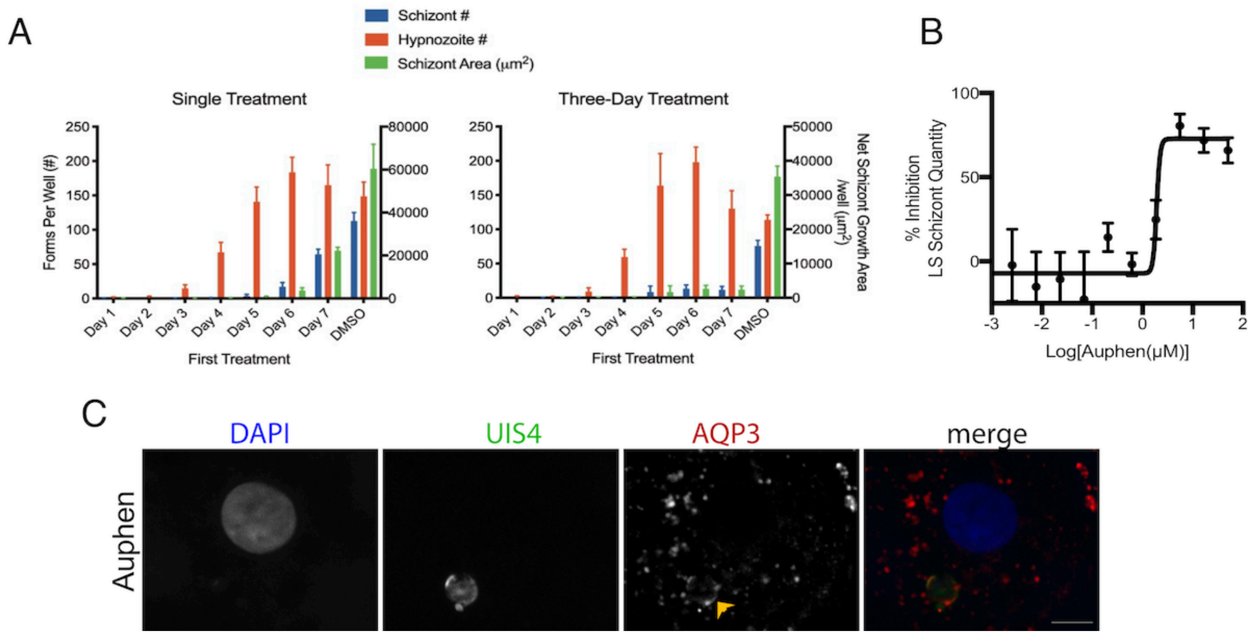

Figure S4, related to Figure 2. Sensitivity to treatment and AQP3 recruitment with auphen. A) PI4K inhibitor MMV390048 was added to *P. vivax* liver stage cultures either once (left) or fresh each day over three days (right), with the first treatment day indicated (x-axis). Number of hypnozoites, number of liver schizonts (left y-axis) and schizont growth area (right y-axis) were quantified by HCI. Errors bars represent SD of triplicate technical replicate wells, data are representative of two independent experiments. B) Dose-response curve of auphen inhibition of *P. vivax* schizont quantity per well. Data shown as the average ± SEM of all independent experiments. C) Confocal images of *P. vivax* LS parasite on day 8 post-infection of PHH in the presence of 1.85 µM auphen. Arrow indicates AQP3 localization following treatment. Cells stained with *Pv*UIS4 (green), *Hs*AQP3 (red), and DAPI (blue). Scale bar = 5 µM.
